# Associations between micro- and macro level social network properties and individual productivity in virtual collaboration

**DOI:** 10.1038/s41598-025-09309-z

**Published:** 2025-07-15

**Authors:** Dongning Deng, Julia Koltai

**Affiliations:** 1 Doctoral School of Sociology, Faculty of Social Sciences, Eötvös Loránd University, Pázmány Péter sétány 1/A, Budapest, 1117 Hungary; 2 Institute of Empirical Studies, Faculty of Social Sciences, Eötvös Loránd University, Pázmány Péter sétány 1/A, Budapest, 1117 Hungary; 3 MTA–TK Lendület “Momentum” Digital Social Science Research Group for Social Stratification, HUN-REN Centre for Social Sciences, Tóth Kálmán utca 4., Budapest, 1097 Hungary

**Keywords:** Social network analysis, Productivity, Teamwork, Collaboration, Github, Digital behavioural patterns, Statistics, Computational science, Human behaviour

## Abstract

Although the connection between social network properties (SNPs) and team productivity has been studied extensively, there is still room to deepen our understanding, particularly regarding individual-level dynamics, the non-linear nature of these relationships, and the interactions between individual and structural factors. To do this, we analysed 58 Open Source Software Development (OSSD) projects, using a comprehensive set of SNPs and measuring individual productivity by code editing contributions. Our findings reveal that SNPs have significant and complex dynamics in their associations with individual productivity. Highly productive individuals present SNP traits with a moderate number of connections, being indirectly connected but having influential peers, and being in a decentralised yet locally cohesive environment. Centralised team structure with direct connection with central nodes or influential clusters benefits individual productivity, especially for those who are peripheral or have powerful peers. The highly productive members in the influential clusters also form and reinforce “coordination chambers”. Low individual productivity or even the free riding phenomenon may be more prevalent in a highly closed local and global environment. This is especially true when the structure is not diverse. Taking on a brokerage role with access to diverse knowledge is generally key to active participation, especially when connections are non-redundant. However, productivity may suffer when individuals become too embedded in the bridging role. To minimise the cost of such brokerage role, how and where to be a broker matters. One can become active either with unique ties in networks with centralised bridging brokerage, decentralised accessibility, or clustered structure, or bridging disconnected groups in less clustered but locally cohesive networks with evenly-distributed influence. Our analytical framework shows how non-linear and contextual interaction dynamics can be uncovered using social network and statistical methods. The findings inform not only how open-source workspaces can be better structured according to governance goals, but also potential inequalities in OSSD teams and a possible approach for more open and inclusive team structures.

## Introduction

Teams are established social units to coordinate individual efforts towards a collective objective. An individual’s productivity in a team can demonstrate complex patterns and dynamics. For example, in larger teams, some individuals may become free-riders and contribute less than others, resulting in the Ringelmann Effect^1^. What leads to variations in productivity among individuals in teams? From a theoretical standpoint, individual behaviour is shaped through ongoing social interactions^2^. As per the social network theory paradigm, both group-level and individual-level Social Network Properties (SNPs) significantly influence productivity^3,4^. The degree of interconnectivity among actors in a network provides access to resources, information, and support, all of which are key drivers of productivity^3,5^. An individual’s position within the network, as well as the network’s overall structure, plays a critical role in shaping their output^3,6–8^. Productivity, viewed as a social outcome, is not merely the result of isolated effort but rather the product of a dynamic process^9,10^. Macro-level factors such as team structures and network configurations influence individual-level behaviours (e.g., collaboration, effort, responsiveness). These micro-level actions, in turn, accumulate to affect higher-level outcomes like team performance and can even reshape the social structure itself.

**Fig. 1:**
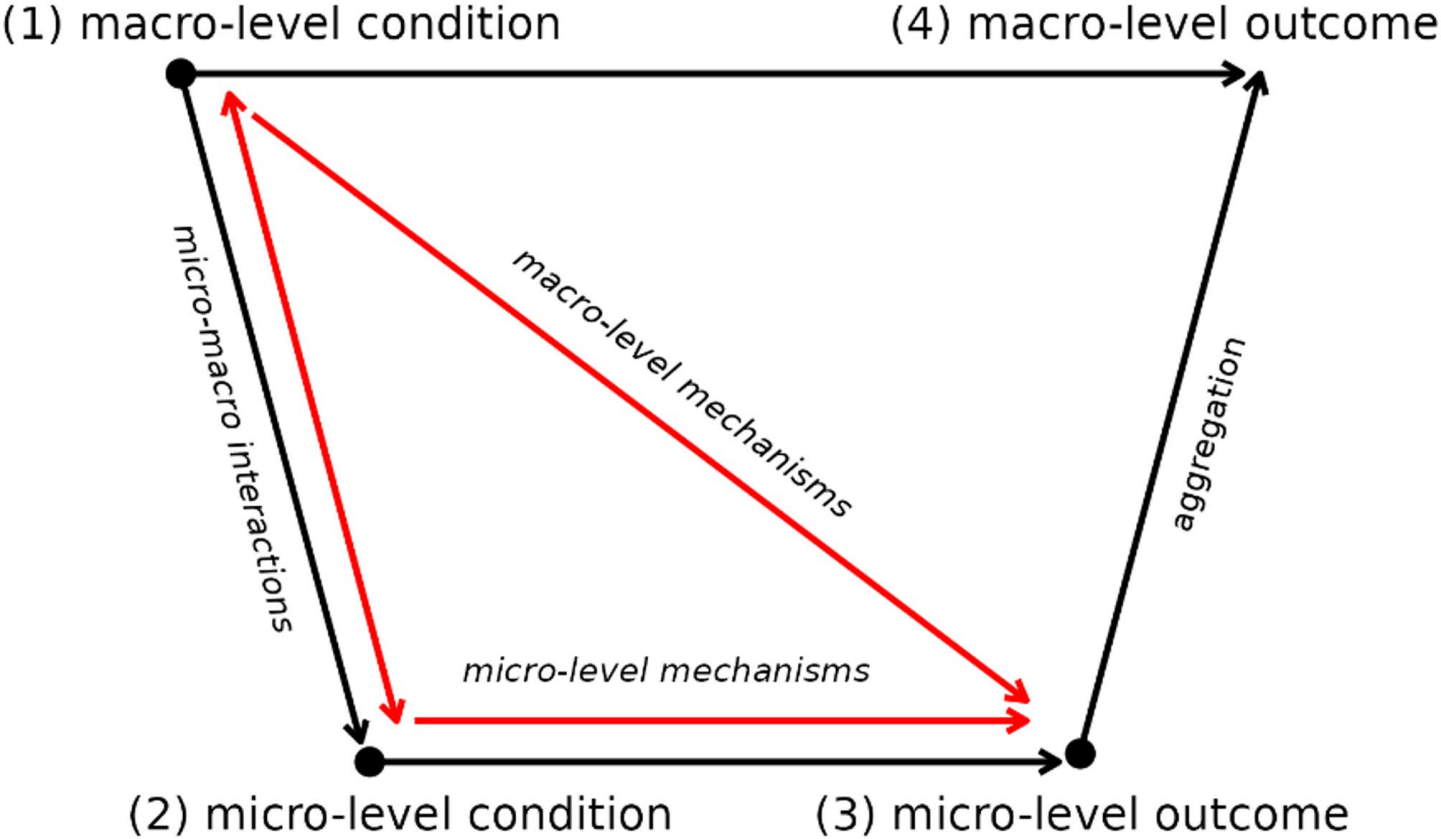
Coleman’s macro-micro-macro model: original and our study focus. Coleman’s Macro-Micro-Macro Model (black lines) illustrates how social structures shape individual actions, which in turn influence collective outcomes. This study focuses on the red-highlighted paths, examining how both macro- (1) and micro-level (2) social network positions, as well as their interaction, relate to individual productivity (3) within teams, addressing less-explored stages of the existing studies.

This study aims to systematically examine the relationship between individual productivity within teams and the structural characteristics of social networks at both the micro and macro levels. To conceptualise this relationship, we reflect on Coleman’s Macro-Micro-Macro Model^11^ ,which provides a macro-micro-macro framework for understanding the interplay between social structures, individual actions and collective outcomes. The original model is visualised as Fig. 1 with black solid lines. The model delineates the following sequence:​ Macro-to-Micro (1 → 2): Social structures influence individual behaviour by shaping the context within which individuals operate; Micro-Level (2 → 3): Individuals make decisions based on the constraints and opportunities presented by their social context.; Micro-to-Macro (3 → 4): Individual actions aggregate to produce macro-level outcomes, potentially reshaping the original social structures. The red lines in Fig. 1 illustrate the theoretical focus in this study. While prior research in OSSD team, as reviewed in the following sections, has primarily concentrated on macro-level outcomes—particularly team productivity as the aggregate of individual outputs—less attention has been paid to the preceding stages of the model. This research seeks to address gaps in the earlier stages of the model by focusing on individual productivity at point 3. Specifically, it investigates the relationship between individual productivity (3) and both macro-level SNPs (1) and micro-level SNPs (2). Furthermore, it explores potential interactions between macro (1) and micro (2) SNPs and how these interactions relate to individual productivity (3).

Coleman’s Macro-Micro-Macro Model (black lines) illustrates how social structures shape individual actions, which in turn influence collective outcomes. This study focuses on the red-highlighted paths, examining how both macro- (1) and micro-level (2) social network positions, as well as their interaction, relate to individual productivity (3) within teams, addressing less-explored stages of the existing studies.

Virtual teams as social entities present valuable potential for exploring this question. The development of digital infrastructure allows for an increasing number of collaborations to be conducted online, and the growing trend of virtual teams has caught the interest of researchers^12–14^. Virtual teams comprise individuals with shared objectives who interact via digital platforms and work without the bureaucratic boundaries typical of traditional organisations^13,15^. A virtual team is considered a unique social entity that houses important information on network structures and agent dynamics^12^. Open Source Software Development (OSSD) teams are a form of entity with unconstrained membership and bottom-up and self-initiated team dynamics^16^. The vast amount of data generated from digital work records provides valuable research opportunities to uncover this information, especially from OSSD teams.

Several studies have demonstrated the important and complex relationship between SNP and virtual teams’ coordination and productivity. It is revealed that there is a significant connection between structural and relational engagement and team coordination and productivity^17^. This highlights how the manner of connections and interactions among virtual teams can impact their success. The correlation between the structure and dynamics of a network and productivity is also explored. Initially, it was hypothesised that a productive virtual team’s social network would display specific features, including high brokerage, moderate closure, and low centrality in leadership^13^. Nevertheless, the empirical data disaffirmed these anticipations, signifying that closure does not associate positively with virtual team productivity, and neither brokerage nor leadership centrality shows significant relationships^18,19^. Additionally, there is research that focuses on specific social network phenomena, for example, the effects of brokers’ appearance on network centralisation and dynamics^20^. It was found that brokerage positively correlates with project productivity. Their example with the process of knowledge transfer shows that more brokers in the team lead to a less centralised environment, which enables the flow of ideas and knowledge transfer. Some other studies have investigated the association between the amount of social connections and personal efficiency. While one study observed a positive relationship between the number of social ties and productivity^21^another study on the Ringelmann effect in OSSD teams found that larger team size correlates with lower individual productivity^22^. The latter study further justifies that such a negative relationship between team size and productivity is linked to the increasing coordination burden in larger teams, diverting each individual’s attention from their own tasks. There are existing but limited studies reporting non-linear relationships between SNPs and productivity. For example, Wu and others concluded the importance of a moderate level of degree centrality in OSSD team structure and software architecture^23^. Pal and others assessed the impact of communication network density on performance and found an inverted U-shaped relationship^24^.

The aforementioned studies highlighted diverse productivity patterns of OSSD teams, subject to varied social network positions and structures. However, research on OSSD teams has excessively prioritised collective productivity, with less emphasis on the effects of individual productivity. Since comprehending individual dynamics can also improve better understanding of team productivity, we will contribute by investigating individual productivity. Generally, the conceptual definition of individual productivity is about the outcome of an individual’s action with a specific organisational purpose^25^. In our case, it is the contribution of an individual member to an OSSD project. It can be analysed from three layers of factors^25^. The initial layer poses two questions regarding whether individual productivity concerns the action aspect, referring to what individuals do at work, or the outcome aspect, relating to the result of their actions. The second layer examines two fundamental dimensions which inquire whether individual productivity is defined by the task dimension (e.g. individuals perform directly for the organisation’s goal) or the contextual dimension (e.g. they perform to support the social environment that is indirectly related to the organisation’s goal). The third layer comprises three perspectives on the primary focus of the definition. One may choose to concentrate on the most efficient individuals, the current situation, or the process of productivity^25^. Out of these factors, we approach individual productivity from the following directions. We begin by concentrating on the outcome aspect of the definition, which implies the result of the work. Subsequently, we assess the contextual dimension of the definition, which means we acknowledge that the OSSD teams tend to perform to voluntarily support the social environment instead of performing directly for any organisation’s goal. While such social support may also contribute to any organisational level of goal, it is rather an indirect connection. Because the motivations behind these voluntary supporters do not originate from any organisational mission, but grow from a need of a social community that provides intrinsic rewards such as intellectual and aesthetic gratification, prestige and visibility for self-development, and collective intelligence for an unfilled market^26^. Thirdly, the focus is on the situation perspective of the definition, investigating which SNPs contribute to enhanced individual productivity. Overall, individual productivity is defined in this paper as the result of an individual’s actions aimed at supporting the social environment. The concept mainly focuses on the situation that influences the outcome. Lastly, we use the individual commit size as a measurement of individual productivity because such action reflects meaningful changes and can be an indicator of effective technical refinement^27^.

Our second contribution is about the coverage of SNPs. Each of the previously mentioned studies possesses a distinctive collection of SNP metrics, influenced by diverse research interests. Typically referenced metrics include individual positions within social networks, such as the broker position and various centralities, alongside network-level metrics, such as network closure, centralisation, and the degree of brokerage in the network. Yet, there has been no systematic testing to ascertain the role of these metrics on individual productivity. We will assist in creating a thorough comprehension of this gap. Thirdly, previous studies appear to lack a systematic investigation into the non-linear relationship of most SNPs on individual productivity. Nor is there any exploration of how the interactions between individual and network SNPs relate to the productivity. Given the complex relationship between SNPs and individual productivity, incorporating these two perspectives helps to provide a clearer and more comprehensive understanding of the underlying dynamics.

## Research questions and hypotheses

This paper aims to investigate the role of different SNPs on the productivity of individuals in virtual teams. The research questions are categorised into three levels: social network positions of individuals, social network structures, and the structure-position interactions:

1. In virtually collaborating teams, how are social network positions of an individual associated with individual productivity?
2. In virtually collaborating teams, how are social network structures associated with individual productivity?
3. In virtually collaborating teams, how are the interactions between network structures and individual positions associated with individual productivity?

We have created nine in-depth hypotheses about the linear relationships between certain SNP dimensions and productivity. We start from a linear relationship, as it has the most prevalent discussion in the existing studies of the topic. In the following, we detail these hypotheses and the related literature based on which we created them. Table 1 provides a summary of the metrics of different levels and concepts used in the hypotheses.

**Table 1:** Social network levels, dimensions and metrics.

	Centrality and centralisation	Closure	Brokerage
Individual level	Degree; closeness centrality; eigenvector centrality	Clustering	Constraint; betweenness centrality
Network level	Degree centralisation; closeness centralisation; eigenvector Centralisation	Clustering; transitivity	Betweenness centralisation

### Centrality and centralisation

It is said that at an individual level, establishing and maintaining a large number of social connections demands extra attention and potentially reduces overall productivity^18,22,28^. As a result, individuals with a larger number of social connections (higher individual degree) may be occupied with managing their relationships and have less energy to complete their own tasks, leading to productivity reduction. Similar dynamics may manifest in OSSD teams. Collaborating in an OSSD project requires significant time and effort as well^18^. For instance, comprehending the assigned tasks could already take a great effort, not to mention the extra attention needed to collaborate and navigate team dynamics. In addition, open-source projects rely on volunteers who have further limited availability. Therefore, an increased amount of interactions is more likely to hinder knowledge creation^28^ and diminish productivity. On the other hand, it is often acknowledged that being in a central social position which connects to many collaborators motivates individuals to contribute^8,29^. Therefore, if an individual holds a leadership role in the team or is in a central position with a significant number of connections, they may be more willing to exert additional effort to enhance their productivity. Based on these discussions on centrality, two hypotheses are formed:

H1. In virtually collaborating teams, having a high number of social ties (degree) is associated with a decrease in individual productivity.

H2. In virtually collaborating teams, having a central position in terms of accessibility to resources (closeness centrality) or influence on others (eigenvector centrality) is associated with an increase in individual productivity.

As the formation of the OSSD team relies on a bottom-up and self-initiated process rather than top-down planning, membership is open to anyone with an interest^16^. Consequently, a flat and decentralised structure constitutes the groundwork of the nature of the OSSD team. This structure is highly favoured by practitioners in the field as it cultivates efficacy and stimulates each member’s contribution^30^. The consideration behind this is that if power is concentrated in one or a few nodes or if the distribution of knowledge is too hierarchical, the flow of knowledge may not be efficient enough to promote creativity - an important element for productivity in the industry^31^and undermine individual productivity. However, this theory is somewhat speculative as in reality, OSSD teams often exhibit some degree of natural centralisation, despite the lack of a predefined centralised structure. For example, they may have an onion-like structure with the most active contributors at the core^30^. Thus, centralisation appears to be a common occurrence in OSSD teams. Then the issue is whether it has adverse effects on personal productivity, as suggested by industry beliefs. Thus, using the industry beliefs as a baseline, the following hypothesis pertains to centralisation:

H3. In virtually collaborating teams, being in a highly centralised team structure in terms of connection distribution (degree centralisation), accessibility to resources (closeness centralisation) or influence on others (eigenvectors centralisation) is associated with a decrease in individual productivity.

### Closure and brokerage

Idea and action are critical components in steering a team towards the desired organisational outcome^32,33^. They are particularly pertinent in knowledge-intensive industries like software development and OSSD teams. In such contexts, novel ideas are needed to catalyse knowledge generation, while the realisation of tangible value hinges upon efficient implementation strategies. Two social network concepts, closure and brokerage, are closely associated with these processes, especially with the idea creation. Briefly, closure can reinforce social connections within a unified group and foster familiarity for improved implementation, but can also stifle creative thinking; while brokerage can enhance external connectivity and facilitate diversity for innovation, but can also hinder idea implementation^33,34^.

In detail, closure refers to the density of a network, questioning whether one’s connections are interlinked with each other and if the overall network is a closed community. Studies indicated that closure fosters strong trust amongst group members but does not promote external communication and creativity implementation^33,34^ - an important element to foster productivity in software development teams^31^. Consequently, in a closed team structure, individual productivity is undermined, which is also indicated in some studies’ findings^18,19^. Closure is often assessed through indicators such as clustering and transitivity, which measure the presence of triadic relationships^35–39^. From these processes, the following hypotheses are formulated:

H4. In virtually collaborating teams, having a high individual clustering level is associated with a decrease in individual productivity.

H5. In virtually collaborating teams, being in a highly clustered team structure is associated with a decrease in individual productivity.

H6. In virtually collaborating teams, being in a high-transitivity team structure is associated with a decrease in individual productivity.

In contrast, brokerage is believed to bring together diverse knowledge sources and enable creativity^33^. Such diversity and creativity, as mentioned earlier, facilitate productivity in software development teams^31^. Common indicators of brokerage are betweenness centralisation, betweenness centrality and individual constraint^30,31,40,41^. At a network level, high betweenness centralisation features the high appearance of brokers who bridge diverse knowledge sources and facilitate information exchange. This can address the previously mentioned issue of inefficient communication flow inherent in centralised network structures^30,31^and potentially improve individual productivity. At the individual level, high in betweenness centrality^41^ or low in individual constraint^40^ signifies the role of being a broker who benefits from diverse knowledge and creativity^33^ and tend to be more productive^31^. All in all, the hypotheses regarding brokerage are:

H7. In virtually collaborating teams, having a low individual constraint level is associated with an increase in individual productivity.

H8. In virtually collaborating teams, having a high betweenness centrality is associated with an increase in individual productivity.

H9. In virtually collaborating teams, being in a team structure with high betweenness centralisation is associated with an increase in individual productivity.

## Data and methods

### Data

The analysis was carried out on a pre-existing dataset from Scholtes and others’ research^22^. The dataset comprises work records of 27,992 developers from 58 GitHub Open Source Software projects – the largest online social coding platform. The data has been made publicly available and is accessible in Scholtes and others’ paper^22^.

The dataset has five key data points: the developers; the names of the files the developers worked on; the developers’ editing amount of each code file, which is calculated by the Levenshtein edit distance^42^ between the historical versions of the code file; when the developer edits this code file; and the name of the project that the developers contribute to. One individual may belong to more than one project.

During data cleaning, we excluded cases with missing data. We also excluded 2,361 developers who work on multiple projects to gain a clear understanding of how network-level properties vary with individual productivity, as well as 1,059 individuals who have never worked with anyone. In the end, we solely concentrated on 24,572 developers, which comprised 87.8% of the sample.

Each row of the resulting data structure comprises information about an individual’s productivity and their related SNP metrics. To compute these metrics for each individual, we first constructed a network for each project. A link between two individuals was established only if they edited at least one file together within the project. Such connections between developers are weighted, meaning that if two developers ever collaborate (edit the same file), the weight of the link between them is the sum of the total times they have collaborated. Social network metrics were then calculated on both the individual and network levels. Individuals belonging to the same project (network) were assigned the same values for network-level characteristics. The calculations were carried out using the R package “igraph”^43^.

### Measures

In the models, the dependent variable is the productivity of individuals. As mentioned in the Data section, Scholtes and others^22^ used the Levenshtein edit distance to calculate the developers’ editing amount for each code file. The Levenshtein distance shows how many single-character edits (deleting, adding, or changing characters) are needed to turn the code before a commit action into the code after the commit^22,42^. In our paper, we took the average of all the Levenshtein edit distances of each individual’s editing history and considered this as their individual productivity.

The independent variables are the weighted individual-level and network-level SNPs presented in Table 1. While there are numerous measures for social network characteristics, we selected the ones that are the most frequently utilised metrics for the SNP aspects of interest. Thoroughly, the notions of centrality and centralisation are linked to three commonly employed SNP terms^44^: individual degree^45^individual centralities^46–50^ and network centralisations^48^. While individual degree is itself a metric, for individual centrality and network centralisation, they can be further broken down to three most common metrics: degree centrality/centralisation (the number of connections), closeness centrality/centralisation (ease of accessibility), and eigenvector centrality/centralisation (influence level)^51^. We exclude degree centrality to avoid redundancy with the individual degree metric. To sum up, the following measures are used in the notions of centrality and centralisation: degree, closeness centrality and eigenvector centrality on the individual level; and degree centralisation, closeness centralisation, and eigenvector centralisation on the network level.

Similar selections are conducted for the metrics for closure and brokerage. The most widespread indicator of closure is the frequent occurrence of triadic relationships^35^where the most commonly utilised metrics are clustering and transitivity^35–39^. Betweenness centrality and centralisation are the two indicators of brokerage, first on an individual level and latter on a network level^41,51^. Additionally, individual brokerage can also be quantified using Burt’s constraint^40,52^. Notably, an individual’s brokerage role can be identified as either high betweenness centrality or low constraint.

### Methods

The analysis was conducted using the R programming language. First, Spearman correlation was utilised to investigate the connection between SNP measures and individual productivity. Then, multilevel regression models were developed that allow for controlling for all other SNP measures, to identify the relationship between SNPs and individual productivity in a more valid way. We differentiated between two levels, one at the individual level and another at the network (project) level.

To explain the variance of individual productivity, we created three base multilevel models with SNPs as independent variables. To investigate the impact of the two-level structure in our data set, we developed two intercept-only models. The first model describes the individual level only (see Eq. (1)), while the second includes project level as random effects, representing a two-level structure (see Eq. (2)).

The independent variables have distinct scales in their original values. To make the results more comparable, we standardised all variables using the Standard Scaling method in R^53^. As SNP measures may have high correlation, in the base models, we thoroughly considered multicollinearity in our models and used the Variance Inflation Factor (VIF) as an indicator of multicollinearity between predictors. While a commonly observed rule is to maintain the VIF below 10^54^, we adhered to a stricter criterion and accepted only values below 5, denoting the absence of multicollinearity^55^. In Model 1, solely independent variables concerning an individual’s social network positions were incorporated (see Eq. (3)). In Models 2 and 3, additional independent variables about social network structures were included (see Eqs. (4) & (5)). Model 2 and Model 3 were separated due to the high multicollinearity when network transitivity and network eigenvector centralisation appear in the same model.

As the literatures did not have consistent findings to the linear relationship between the SNPs and individual productivity, we created twelve quadratic models to test the non-linear relationships (see Eq. (6)). The quadratic models are based on base models 2 and 3. In each quadratic model, only one SNP quadratic term was added (Appendix C1, C2).

Furthermore, to have a more nuanced understanding of the relationships, we tested the interaction effects between the individual- and network-level variables on productivity. To have a clear interpretation of the effects, we added the interaction terms one by one to the basic models 2 and 3 (Appendix D1, D2, D3, D4).

## Results

The analysis used a public dataset from Scholtes et al.^19^comprising records from 27,992 developers across 58 GitHub projects. The dataset includes developer names, work file names, file edits (measured by Levenshtein distance), edit event time, and project names. We focus on developers working on a single project, which represented 87.8% of the original sample. After data cleaning, 24,572 individuals remained.

**Fig. 2:**
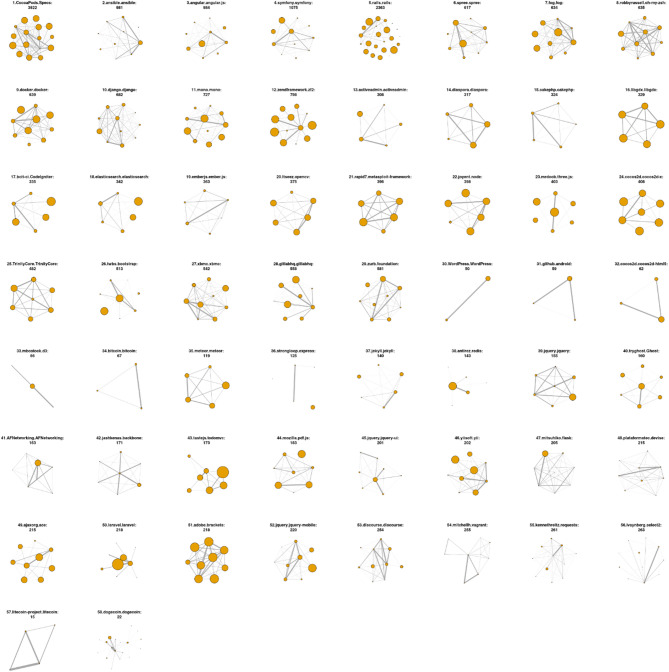
OSSD team network visualisation. Connectivity among important nodes in each OSSD project. For better visualisation, we filter the nodes by degree and edges by the frequency of interactions in case of high-density graphs. In the visuals, only the top nodes (top 1–4%) and edges (top 10% - all) are shown. Each graph represents a specific OSSD project, with nodes indicating individuals, and edges showing their connections defined by collaborating on the same files. All nodes have sizes by productivity. All edges have weights by the frequency of interactions. The titles are project names, with the project’s member size showing the total original number of developers in the same project. The graphs show various network structures in the projects, including star-shaped networks (e.g., plots 28, 40, 42 and 50), fully connected cliques (e.g., plots 10, 14,21, and 57), and sparse linear structures (e.g., plots 30 to 33). Some projects display a community structure with modular teams (e.g., plot 43, 54, 58), trio structures (e.g., plot 34), and hierarchical tree-like networks (e.g., plot 45). The graphs also display different levels of centralisation structures by productivity. In most cases, the central nodes are the most productive ones. But how the connection is distributed by the productive nodes varies by project. Some projects have multiple highly productive central nodes (e.g. plots 1,16, and 24). Some have the connections concentrated on a few highly productive nodes (e.g. plot 2, 26 and 50). Some projects have central nodes that are significantly less productive than those in the other projects (e.g. plots 54 and 56). These patterns highlight different collaboration dynamics, from centralised to distributed, reflecting how the top contributors interact and organise their work in the OSSD projects.

Figure 2 provides a general overview of the connectivity among the main nodes in the 58 OSSD projects. The descriptive statistics of the variables that are included in the regression analysis are displayed in Appendix A.

### Connectivity among important nodes in each OSSD project

For better visualisation, we filter the nodes by degree and edges by the frequency of interactions in case of high-density graphs. In the visuals, only the top nodes (top 1–4%) and edges (top 10% - all) are shown. Each graph represents a specific OSSD project, with nodes indicating individuals, and edges showing their connections defined by collaborating on the same files. All nodes have sizes by productivity. All edges have weights by the frequency of interactions. The titles are project names, with the project’s member size showing the total original number of developers in the same project. The graphs show various network structures in the projects, including star-shaped networks (e.g., plots 28, 40, 42 and 50), fully connected cliques (e.g., plots 10, 14,21, and 57), and sparse linear structures (e.g., plots 30 to 33). Some projects display a community structure with modular teams (e.g., plot 43, 54, 58), trio structures (e.g., plot 34), and hierarchical tree-like networks (e.g., plot 45). The graphs also display different levels of centralisation structures by productivity. In most cases, the central nodes are the most productive ones. But how the connection is distributed by the productive nodes varies by project. Some projects have multiple highly productive central nodes (e.g. plots 1,16, and 24). Some have the connections concentrated on a few highly productive nodes (e.g. plot 2, 26 and 50). Some projects have central nodes that are significantly less productive than those in the other projects (e.g. plots 54 and 56). These patterns highlight different collaboration dynamics, from centralised to distributed, reflecting how the top contributors interact and organise their work in the OSSD projects.

Table 2 demonstrates the correlations between the variables, indicating a significant correlation between most independent variables. For instance, the strongest positive correlation is observed between network betweenness and eigenvector centralisation, while network transitivity and eigenvector centralisation show the strongest negative correlation. According to the correlations, individually, those with high clustering and constraint are related to a lower level of individual productivity. Those with a higher value of degree, betweenness and eigenvector centrality are related to a higher individual productivity. At the network level, a negative correlation is seen between high levels of clustering or centralisation (betweenness, closeness, eigenvector and degree) and individual productivity, and a positive correlation is shown between high values of network transitivity and individual productivity.

**Table 2:** Spearman correlations between variables.

	1	2	3	4	5	6	7	8	9	10	11	12	13
1. Individual productivity	-												
2. Individual-level clustering	-0.30***	-											
3. Individual-level degree	0.09***	-0.12***	-										
4. Individual-level constraint	-0.06***	-0.12***	-0.02***	-									
5. Individual-level betweenness centrality	0.10***	-0.19***	0.02**	-0.07***	-								
6. Individual-level closeness centrality	-0.01	-0.15***	-0.00	0.11***	-0.01	-							
7. Individual-level egenvector centrality	0.24***	-0.37***	0.28***	-0.05***	0.16***	-0.00	-						
8. Network-level clustering	-0.03***	0.11***	-0.03***	0.05***	0.03***	-0.01	-0.01	-					
9. Network-level transitivity	0.08***	0.01	0.04***	0.11***	-0.07***	0.02*	0.11***	-0.06***	-				
10. Network-level betweenness centralisation	-0.06***	-0.04***	-0.04***	0.20***	0.06***	0.04***	-0.00	0.37***	-0.52***	-			
11. Network-level closeness centralisation	-0.07***	-0.03***	-0.04***	0.10***	0.00	0.06***	-0.04***	-0.17***	-0.41***	0.08***	-		
12. Network-level eigenvector centralisation	-0.09***	-0.03***	-0.05***	-0.12***	0.06***	0.00	-0.11***	-0.00	-0.95***	0.55***	0.46***	-	
13. Network-level degree centralisation	-0.03***	0.04***	-0.02*	0.10***	0.03***	-0.07***	-0.12***	0.02**	-0.55***	0.20***	0.34***	0.49***	-

***p < 0.001; **p  <0.01; *p < 0.05.

To gain a thorough understanding of the link between each SNP and individual productivity, we carried out additional multilevel regression analysis with other SNP measures controlled. Noted that all the social network properties are calculated on the weighted networks, where weights are equal to the number of files to the developers edited together.

Table 3 indicates that the intercept model, which accounts for the two-level nature of the data, exhibits a considerably better fit compared to the intercept model that only relates to the individual level (*x*^2^ = 1712.3, p *<* 0.001). Consequently, it is recommended to consider the two-level structure in further complex models. Table 3 shows the results of the intercept-only models. It is indicated that 9% of the variation in productivity can be accounted for by project-level factors, while 91% can be attributed to individual-level factors.

**Table 3:** Results of multilevel regression: Intercept-only model.

	Estimates	std. error	CI	*p*	df
(Intercept)	0.03	0.04	-0.06–0.10	0.537	24569.00
Random effects					
*σ* ^2^	0.93				
*τ*_00_ (*Project*)	0.09				
ICC	0.09				
N (*Project*)	58				
Observations	24,572				
Marginal R^2^ / Conditional R^2^	0.00/0.09				

Table 4 shows the detailed multilevel regression results between the SNPs and individual productivity. The multicollinearity range in these analyses lies between 1.00 and 4.36. When compared to the model relying solely on the intercept, incorporating individual social network measures as predictors in Model 1 exhibited significantly better fit for the data (Model 1: *x*^2^ = 3348.8, p *<* 0.001).

Furthermore, including network-level SNP measures in the model led to a slight increase in the explained variance. Model 2 and Model 3 show slightly better fits compared to Model 1 (Model 2: *x*^2^ = 9.6632, p *<* 0.1; Model 3: *x*^2^ = 11.021, p *<* 0.1). The Marginal R-squared statistics for Models 1 to 3 indicate that our predictors account for 13% of the variation in individual productivity.

Except for individual degree, all other SNP measures at the individual level have consistently significant associations with individual productivity. Individuals with high values of clustering, constraint, and closeness centrality demonstrate lower productivity, whereas those with high betweenness- and eigenvector centrality display increased productivity. Notably, the eigenvector centrality measure has the most positive association, and the clustering measure has the most negative association. These effects exhibit stability across all models. Neither the directions nor the significance of the individual-level SNP measures changed with the inclusion of network-level variables in Model 2 and Model 3. In the network-level SNPs, Models 2 and 3 both show that a network with higher degree centralisation is related to higher individual productivity. The remaining network-level predictors do not have a significant association with individual productivity. It is important to emphasise that these are results that can be interpreted ceteris paribus, so the effects of the SNP indices are present while we control for all other SNP indices included in the model.

**Table 4:** Results of multilevel regression models with individual productivity and SNP Predictors.

	Base models		
	Model 1	Model 2	Model 3
Intercept	0.04	0.09	0.07
	(0.04)	(0.08)	(0.08)
Individual clustering	-0.27***	-0.27***	-0.27***
	(0.01)	(0.01)	(0.01)
Individual degree	0.01	0.01	0.01
	(0.01)	(0.01)	(0.01)
Individual constraint	-0.13***	-0.13***	-0.13***
	(0.01)	(0.01)	(0.01)
Individual betweenness centrality	0.03***	0.03***	0.03***
	(0.01)	(0.01)	(0.01)
Individual closeness centrality	-0.02***	-0.02***	-0.02***
	(0.01)	(0.01)	(0.01)
Individual eigenvector centrality	0.12***	0.12***	0.12***
	(0.01)	(0.01)	(0.01)
Network clustering		0.01	0.01
		(0.04)	(0.04)
Network transitivity		0.09	
		(0.06)	
Network betweenness centralisation		-0.27	-0.21
		(0.31)	(0.31)
Network closeness centralisation		-0.02	0.01
		(0.04)	(0.05)
Network degree centralisation		0.12*	0.12**
		(0.05)	(0.04)
Network eigenvector centralisation			-0.11
			(0.06)
AIC	64742.25	64761.87	64760.73
BIC	64815.24	64875.40	64874.26
Log Likelihood	-32362.13	-32366.94	-32366.36
Num. obs.	24,572	24,572	24,572
Num. groups: project	58	58	58
Var: project (Intercept)	0.10	0.09	0.09
Var: Residual	0.81	0.81	0.81
Marginal R^2^ / Conditional R^2^	0.13/0.22	0.13/0.22	0.13/0.22

***p < 0.001; **p  < 0.01; *p  < 0.05.

**Fig. 3:**
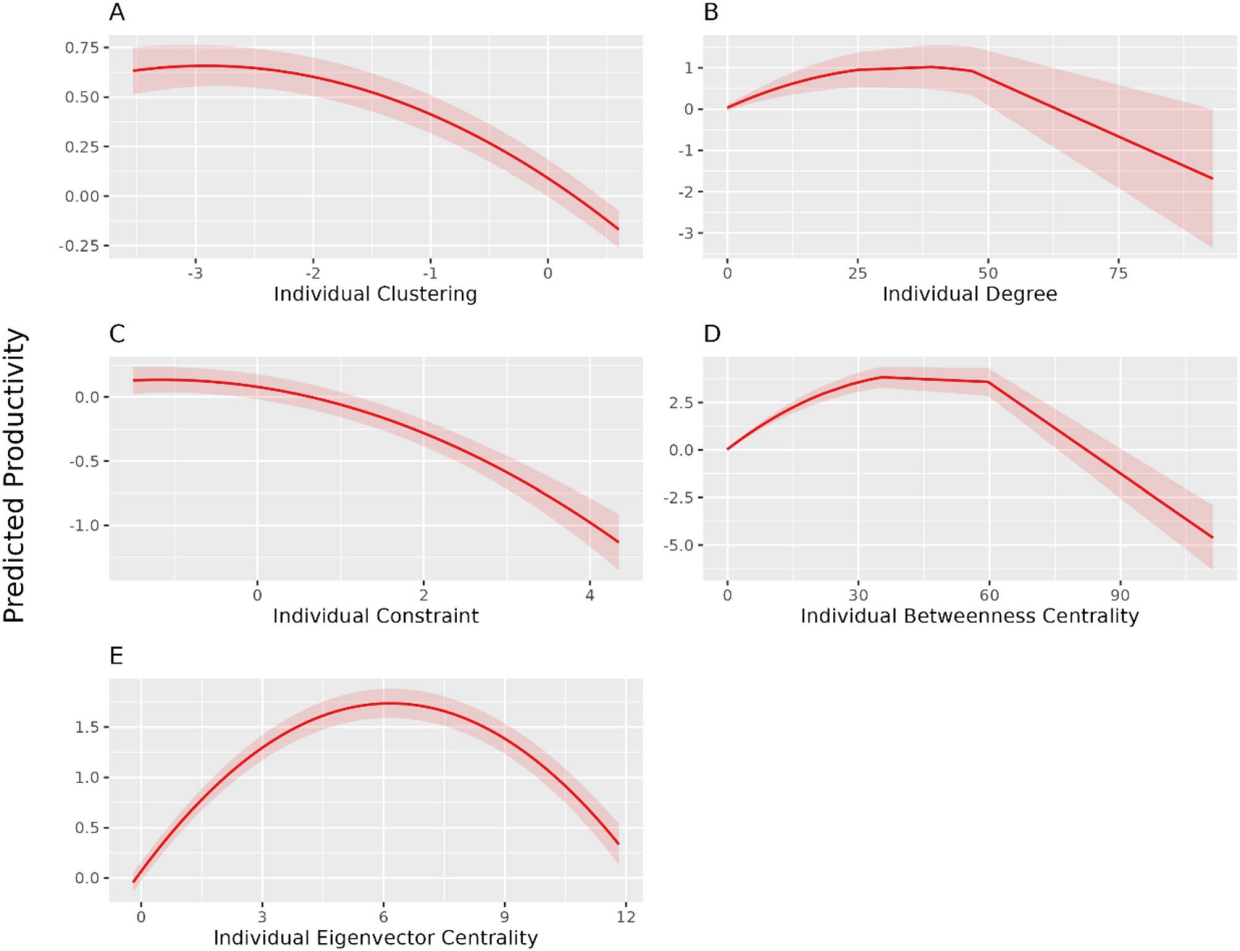
Significant non-linear association of individual SNPs and predicted productivity. Significant non-linear relationships between SNPs and productivity. All the variables in the plots are in their scaled values. The network SNPs do not show any significant non-linear relationships with the productivity. Higher individual clustering (**A**) or individual constraint (**C**), especially when the value exceeds the mean, is associated with reduced productivity. Individual degree (**B**), betweenness centrality (**D**) or Eigenvector centrality (**E**) all show an inverted U-shaped relationship, suggesting that moderate connectivity, levels of brokerage or intermediate levels of influence within the network maximise productivity.

Figure 3 represents the significant results of the SNP quadratic models. The detailed model components are in Appendix C1–C2. Overall, the significant non-linear relationships are all shown with the individual SNPs. The network SNPs are either not significant in both linear and quadratic terms, or significant in only the linear terms (e.g. eigenvector centralisation or network transitivity). Therefore, we focus on the results among the individual SNPs. The individual clustering and constraint have accelerating negative relationships with the individual productivity - their effects become increasingly detrimental beyond certain thresholds. The individual degree, betweenness- and eigenvector centrality have inverted U-shape relationships with the individual productivity, suggesting an optimal range of centrality for maximising individual productivity, after which the benefits decline.

### Significant non-linear relationships between SNPs and productivity

All the variables in the plots are in their scaled values. The network SNPs do not show any significant non-linear relationships with the productivity. Higher individual clustering (A) or individual constraint (C), especially when the value exceeds the mean, is associated with reduced productivity. Individual degree (B), betweenness centrality (D) or Eigenvector centrality (E) all show an inverted U-shaped relationship, suggesting that moderate connectivity, levels of brokerage or intermediate levels of influence within the network maximise productivity.

**Fig. 4:**
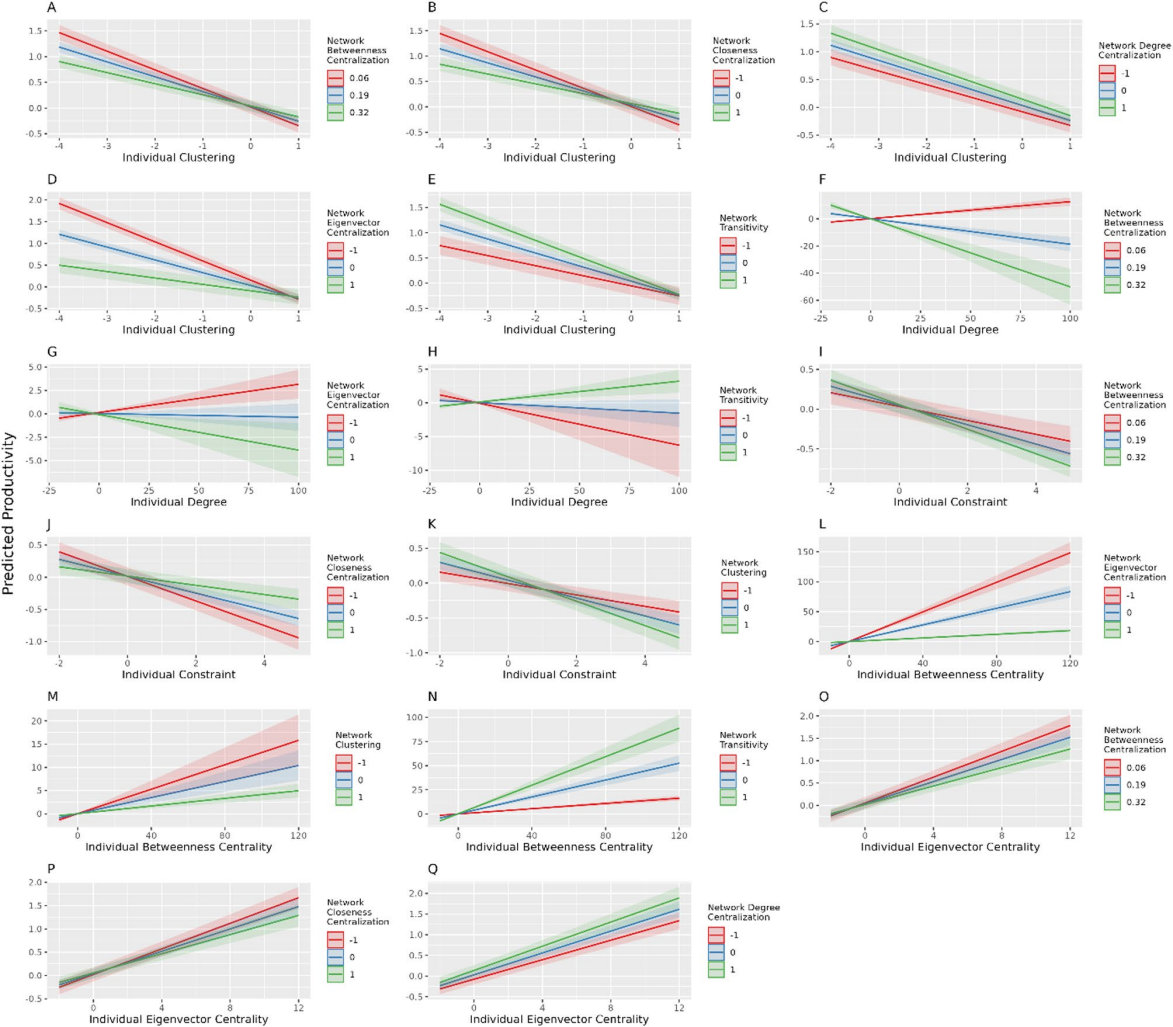
Significant interactions between individual and network SNPs – individual level perspective. Certain network-level SNPs significantly moderate the relationship between some individual SNPs and productivity. All the network-level SNPs are categorised by mean and SD of the scaled values. Plots **A** to **E** show that the negative relationship between individual clustering and productivity changes by the levels of network centralisation and transitivity. Plot **F** to **H** shows that the association between individual degree and productivity changes dramatically by the levels of network betweenness-, eigenvector centralisation or transitivity. Plots I to K demonstrate the moderation of network betweenness-, closeness centralisation and clustering to the negative relationship between individual constraint and productivity. Plot **L** to **N** demonstrate that the positive association between individual betweenness centrality and productivity is moderated by the levels of network-level eigenvector centralisation, clustering or transitivity. Plot **O** to **Q** presents the moderation of multiple network centralisations to the positive association between individual eigenvector centrality and productivity.

To investigate the interaction effects between the individual- and network-level predictors, we configured 31 models with interaction variables based on the base models containing individual and network SNPs (Models 2 & 3 in Table 4). Figure 4 summarises the significant interactions in the models (Full results see Appendix D1, D2, D3, D4). From an individual SNP perspective (Fig. 4), except for individual closeness centrality, other individual SNPs’ effects are moderated by different network-level predictors. The negative association between individual clustering and productivity is stronger when the betweenness-, closeness-, and eigenvector centralisation is the lowest, or when the degree centralisation or network transitivity is the highest. For network-level betweenness- and closeness centralisation, the effects are the weakest when the individual clustering value is near its mean. For others, the effects of network-level metrics get smaller when the individual clustering value gets higher (Fig. 4 plots A–E). The association between individual degree and productivity is positive when network betweenness or eigenvector centrality is the lowest, or when network transitivity is the highest. The effects of network-level metrics get stronger when the individual degree is above its mean (Fig. 4, plot F to H). The association between individual constraint and productivity is the most negative when the network betweenness or clustering is the highest, or when network closeness is the lowest. The network-level effects are minimal when the individual constraint is slightly above its mean (Fig. 4 plots I to K). The association between individual betweenness centrality and productivity is the most positive when network-level eigenvector centralisation or clustering is the lowest, or when transitivity is the highest. The network-level effects are the smallest when the individual betweenness centrality is near or slightly above its mean (Fig. 4, plots L to N). Similarly, the association between individual eigenvector centrality and productivity is the most positive when network betweenness or closeness is the lowest, or when network degree centralisation is the highest. For network betweenness and closeness, their effects are the smallest when the individual eigenvector centrality is near its mean (Fig. 4, plot O to Q). Except for individual degrees, all other individual-network SNP interactions are at their minimum when the corresponding individual SNPs are near their mean values.

Certain network-level SNPs significantly moderate the relationship between some individual SNPs and productivity. All the network-level SNPs are categorised by mean and SD of the scaled values. Plots A to E show that the negative relationship between individual clustering and productivity changes by the levels of network centralisation and transitivity. Plot F to H shows that the association between individual degree and productivity changes dramatically by the levels of network betweenness-, eigenvector centralisation or transitivity. Plots I to K demonstrate the moderation of network betweenness-, closeness centralisation and clustering to the negative relationship between individual constraint and productivity. Plot L to N demonstrate that the positive association between individual betweenness centrality and productivity is moderated by the levels of network-level eigenvector centralisation, clustering or transitivity. Plot O to Q presents the moderation of multiple network centralisations to the positive association between individual eigenvector centrality and productivity.

Table 5 summarises all the results from the above models. Overall, the findings in the base models suggest that social network properties have important connections with individual productivity, especially individual SNPs as most of them have significant results. Results in the quadratic models and interaction models reveal that beneath the linear relationships there are complex dynamics as the connections are not all linear and the structures interact with individual positions.

**Table 5:** Summary of results in base (Linear), quadratic (Non-linear) and interaction models.

Predictors				Centralisation			Closure		Brokerage	Network level
				Degree centralisation	Closeness centralisation	Eigenvector centralisation	Clustering	Transitivity	Betweenness centralisation	
Individual level		Linear		+	/	/	/	/	/	Linear
			Non-linear	/	/	/	/	/	/	Non-linear
Centrality	Degree	/	Inverted U	x	/	−	/	+	−	Interactions
	Closeness centrality	−	/	/	x	/	/	/	/	
	Eigenvector centrality	+	Inverted U	+	−	x	/	/	−	
Closure	Clustering	−	−	−	+	+	x	−	+	
Brokerage	Constraint	−	−	/	+	/	−	/	−	
	Betweenness centrality	+	Inverted U	/	/	−	−	+	x	

“−”: negative relationship. “+”: positive relationship. “/”: not significant relationship. “x”: not included as belonging to the same theoretical concept.

## Discussion

### Centrality and centralisation

Centrality is often considered an indication of leadership, popularity, and reputation^56^. Generally, studies imply that central individuals tend to be more productive^8,29^. Our study provided detail insights to such phenomenon based on various measures of centrality and revealed mix patterns between individual centrality and productivity.

First, being central can be reflected by the number of social connections one has, measured by individual degree. Previous studies reach no agreement on whether being central with a significant amount of social connections benefits or hinders individual productivity^18,21,22,28^. Using identical data from Scholtes and others^22^we found an inverted U-shaped relationship here, which challenges both their conclusion of a negative relationship due to coordination burden and our Hypothesis H1. The U-shape indicates that individual connections are related to higher individual productivity until they reach a certain size of frequency. This supports the findings in some studies about the quadratic effect of social connections to productivity in OSSD team^23,24^. The difference in the results could be due to the different analysis approach. Scholtes and others prioritised examining the linear correlation between team size and individual productivity, with individual-directed degree serving as an explanatory variable in their log-transformed linear regression models. Our study used the individual’s undirected degree and controlled for other SNP measures in our multilevel regression models. Moreover, there is a data coverage difference between the studies. We analysed 58 projects while Scholtes and others included only 48 projects.

Besides confirming the U-shaped dynamic in the existing degree-productivity discussion, our interaction models further extended the discussion. The direction change in the degree-productivity connection can be moderated by certain network structures. Having more connections benefits individual productivity when the network is less centralised, where influence or resources are distributed more equally among members or locally more cohesive with closed triads. It becomes less beneficial when the network is more centralised or lacks local cohesiveness.

Second, a central position can also be reflected as the way one is connected in the network. We found that the individual centrality-productivity association depends on the types of central position. In terms of central individuals who can efficiently access or be reached by others within a network, they tend to have lower productivity. The quadratic model did not reject such a linear relationship, as there is no significance in the squared term of individual closeness centrality. This rejects the positive trend assumed in hypothesis H2. The relationship is not affected by the network context. The coordination burden mentioned earlier about individual degrees could have a role here. In the context of OSSD, individuals come from a diverse talent pool where there are varying coding styles and complexities. When individuals are central and easy to connect with, they are more likely to collaborate with diverse collaborators and may invest more time and effort to consolidate the different coding styles and complexities. Therefore, it results in individual productivity loss.

Individuals who occupy central positions that enable collaboration with influential members tend to be more productive until such centrality surpasses a certain threshold. This rejects the linear assumption in hypothesis H2. In other words, while it is important to work in a supportive environment with access to well-connected peers^57^there is also a drawback of being too embedded in highly influential circles, possibly due to information overload, coordination challenges with the influencers, or even “free-rider” incentives of the individuals. The benefits of individual eigenvector centrality are also amplified in specific network contexts. In networks where brokerage roles or direct paths are limited, or coordination flows through a few highly connected central nodes, indirect influence through powerful ties becomes more valuable. In such environments, having influential connections and alignment with power hubs seems to compensate for a lack of direct control over information flow, enhance access to key resources and decision-makers, and thus benefit individual productivity.

Regarding network centralisation, there are mixed findings in the existing literature. Some studies report no association^18,19^others suggest that high levels of centralisation are detrimental^18,20,58,59^or a moderate centralisation may be ideal^59^. These studies focus on group productivity rather than individual productivity, and their definition of centralisation did not specify centralisation types. We fill this gap by focusing on individual productivity and including detailed centralisation measures to unearth distinct trends. Unlike individual centralities, the significant associations of centralisations with productivity are generally linear, as the quadratic models do not indicate any non-linear relationships in these metrics. In the linear models, the only significant centralisation metric is degree centralisation, which is positively related to higher individual productivity. High degree centralisation signifies a network that has connections centred by one or a few nodes with high connections^48,56^. In such an OSSD team structure, individuals coordinate directly with a few central nodes and, on average, handle less diverse coding styles and complexities. Thus, individual productivity could be optimised in general. However, such benefits of the centralised structure vary by an individual’s local situation. The interaction models indicate that the structure is more beneficial for those who are not clustered in a closed circle or those who are well-connected to the influential contributors. As for network eigenvector centralisation, while it is not significantly related to the productivity in the linear model, it shows a significant relationship in the interaction models when individual SNP moderators are included. High eigenvector centralisation represents a centralised network where influence is heavily concentrated in a few nodes^60,61^. Our interaction model shows that this structure benefits individual productivity more for those clustered in a closed circle or who are not brokers, and diminishes it more for those with a large number of connections. While there is no existing discussion in this interaction effects related to the OSSD team, the findings can be linked to a general discussion about how high eigenvector centralisation fosters echo chambers and skews the influence on network consensus. Bienenstock and Bonacich studied this phenomenon with simulated data in terms of information diffusion in a business organization^60^. Our findings may imply that similar “coordination chambers” exist in the OSSD team coding activity. Members within influential, tightly-knit clusters become more productive due to stronger internal alignment in coding styles, which results in closer interaction within the cluster and reinforces the coordination chamber.

Overall, our findings on Centralisations reject the Centralisation hypothesis (H3) which stated overall negative relationships between the different Centralisations and individual productivity. These findings extended to the ongoing Centralisation-productivity discussion about OSSD team by distinguishing the specific types of centralisations and opened new ideas with the interactions between network- and individual level SNPs.

### Closure

Clustering is a phenomenon where individuals with similar traits tend to be in close proximity^62^. In the theory of the strength of weak ties^63^clusters are described as strong ties with overlapping knowledge and less exclusive information^63,64^. As a result, clusters impede productivity due to a lack of inspiration from external stimuli^64^. Whilst efficient knowledge flows and a high level of trust exist within each cluster, there is greater isolation and difficulty regarding information transfer between clusters^64^. Knowledge-intensive industry, such as OSSD, is reliant on diverse expertise and knowledge^31^ and thus, external information is invaluable for individual developer productivity. In our hypothesis on closure, productivity is negatively related regardless of how it was measured – whether at the individual level clustering (H4), network level clustering (H5), or transitivity (H6).

The results in base and quadratic models confirmed the hypotheses H4, but rejected H5 and H6 as no significant association. Overall, when an individual’s collaborators also collaborate closely with one another, forming a tightly-knit clique, the individual tends to show lower contribution levels. The quadratic model further reveals that this negative relationship is not constant: the greater the integration within a collaboration clique, the less likely individuals are to participate. However, the interaction models shed more complex dynamics as the closure-productivity relationship turns out to be moderated by some network SNPs. The negativity is stronger when one is in a network structure which is decentralised in terms of brokerage, accessibility or influence, or centralised by connection amounts in a few nodes, or a network with more cohesive subgroups where nodes are highly interconnected. These patterns suggest that the broader network context plays an important role in shaping how local clustering relates to contribution behaviour. In decentralised networks that are less structurally diverse or those with fragmented yet highly connected hubs, being embedded in a tightly-knit collaboration clique may become more restrictive for the individuals’ participation.

These findings generally align with the prior research^18,19^ about the negative clustering-productivity relationship in virtual software development collaborations. Our contributions here are not only confirming this association pattern through a different measure of closure, using clustering rather than density as in the previous studies from Hinds^16^ and Vreugdenhil^19^but also adding more insights with the non-linear dynamics as well as the moderation effects from different network structures. We show the importance of information exchange within the industry and underscore the need to consider both local and global network configurations when aiming to improve individual participation.

### Brokerage

Brokerage is generally found to enhance the team^59,65^ and individual^66^ productivity due to the accessibility of diverse information and knowledge. We hypothesised this with various measures of brokerage. A low constraint value (H7), which means having fewer redundant social ties^40^or a high betweenness centrality (H8), which means being a bridge between disconnected nodes and groups^47,67,68^can both signify the individual as a broker and is expected to associate with a boost in their productivity. Specifically, Burt’s constraint measure computes brokerage considering the local neighbourhood environment of a node, while Freeman’s betweenness centrality is more a sociometric measure focusing on the global traffic in the whole network^69,70^. A network structure with high betweenness Centralisation, which centralizes brokerage, is expected to associate with enhanced individual productivity (H9). The hypothesis H9 is rejected in all the models as there is no significant association. The below discussion will focus on the individual brokerage roles.

The base model results supported both hypotheses H7 and H8, even though they used two different brokerage measures. In general individual’s brokerage role is positively linked to active participation in the code editing. However, the quadratic and interaction models inform more complex dynamics about the two brokerage measures. First, both measures’ associations with productivity are not linear, and the shape of the non-linearities is not the same. For constraint, being a broker with fewer redundant ties is overall beneficial to individual productivity, and the productivity increase accelerates as redundancy decreases further. On the other hand, for betweenness centrality, the relationship with individual participation exhibits an inverted U-shape. At lower levels of betweenness, where individuals play a light brokerage role by occasionally bridging others, participation in code editing tends to increase. This positive association peaks at a moderate level of betweenness centrality. As individuals become more heavily embedded in brokerage roles—frequently acting as bridges between others—their participation begins to decline.

Second, the embedded network structures moderate such brokerage roles to individual productivity. Different forms of brokerage respond differently to varying network structures. Being a broker with fewer redundant connections may boost individual productivity in a network where the bridging brokerage role is more centralised, or accessibility is decentralised, or is more clustered as a tight-knit group. Taking a broker role by bridging disconnected groups may boost individual productivity when the network’s influence is more decentralised, or is less clustered as a tight-knit group, but locally more cohesive with closed triads.

To summarise, we not only confirmed the positive association between brokerage and productivity, but also provided new details on how different forms of brokerage affect the individual’s tendency to participate in code editing. Reducing redundancy or strengthening the bridging position in collaboration generally may motivate individuals to participate, but the magnitude of the benefits varies by the structural context. Further specifications in the type of brokerage measure and consider the structural context matter to the analysis. Our discoveries also open a conversation about whether taking a brokering role comes with a cost. That is, whether the need to manage redundant contacts and bridge diversities^67^ result in an adverse effect on their productivity in independent tasks, especially in a certain structural context.

### Implications

The first implication is about the framework of using social network methods in analysing digital collaborative activities. We provided valuable examples of how detailed and complex dynamics can be discovered within the non-linear relationship between social network positions and productivity, and showed that the effects of individual network positions on productivity can vary under different network structures and circumstances.

Second, the findings offer practical implications in how collaborative digital workspaces, particularly in open-source environments, could be structured and managed. As virtual teams increasingly become the norm, particularly in domains like OSSD, these results underscore the importance of not only who the individuals are connected to, but also how they are embedded within broader digital social structures. We specifically address the coding activity dynamics in terms of edit amount. Depending on feasibility, managers can adjust individual and/or structural properties to encourage different scenarios: it can be motivating individuals to participate in more diverse tasks, or it can be reducing individual workload or the complexity of their daily work. For example, positioning core members with moderate centrality to reduce overloading; growing local triads to prevent free riding; centralising the coordination to reduce overall complexity but have a balanced global and local closure; not only spotting the brokers but also identifying their brokerage types to position them in the right team for thriving.

The findings also reveal the potential inequalities in OSSD teams. Previous research indicates that open-source communities often exhibit disparities in contributor participation, with a small percentage of individuals accounting for the majority of contributions^71^. In our results, the Matthew Effect^72^ may be reflected in the “coordination chamber”. For example, those positioned advantageously within the network may have outsized opportunities and influence, while others may be structurally constrained despite similar levels of effort or skill, and their incentives can be reduced. Since the initial idea of OSSD is to leverage the opportunity for voluntary contributions from a wide range of talents and encourage collaborative participation and open exchange^73,74^these structural inequalities may undermine the essential principles of inclusivity and equal opportunity.

### Limitations

We would also like to address the endogeneity problem potentially present in the study. We started from the theoretical standpoint that social network properties affect individual behaviour^75^. However, we have to note that the relationship between social network properties and individual behaviour is not exogenous: accordingly, we analysed the results in terms of association rather than causality. Generally, having a causal inference of the social network effect on individual behaviour is a key challenge in empirical social network studies^76^. While our study did not focus on investigating the causality of the relationship – especially the potential reverse causality when individual productivity affects social network positions –, and because of data limitations we could not even test is, we acknowledge that the investigation of endogeneity could provide in-depth insights into the dynamics between network characteristics and individual productivity.

There are other limitations in this study. First, productivity is a multifaceted phenomenon influenced by various types of social factors. Due to limited data, wider factors such as individual workload, task difficulty, skillsets, developer experience, demographics and personality cannot be captured in the analysis. The omitted variable bias can confound individual motivations, external incentives and the observed relationships. Future studies could aim to control for some of these factors if data is available. Second, the measurement of productivity is limited due to data availability. The representation of editing distance cannot cover all the aspects of productivity. For example, some qualitative aspects of individual contribution, such as code complexity and debugging efficiency, could provide more in-depth insights on how productive an individual is. Future studies exploring alternative productivity measures can provide valuable insights into the SNP-productivity relationship. Third, we were not able to assess work quality with our productivity concept. Perhaps in some cases, a high editing distance may not be preferred because it can show inefficiency. In these cases, methods to reduce editing distance, i.e. reduce the “productivity” defined in this study, may be preferred. Thus, the reverted conclusions would be drawn from this study, for example, reducing the chances of individuals being brokers to reduce the “productivity”. If future studies enrol other measures that reflect not editing amounts but coding efficiency, there will be different dynamics. Lastly, our conclusions are based solely on a snapshot of individual productivity and SNP status at the end time when the project data was collected. However, we also acknowledge that individuals and teams may benefit from innovation-oriented structures at one stage and delivery-focused structures at another. Therefore, a further investigation of the evolution of the SNPs and the edit distance over time could offer additional insight into the temporal dynamics between the network characteristics and individual productivity.

## Data Availability

The data that support the findings of this study are available at: Scholtes I, Mavrodiev P, Schweitzer F (2015) From aristotle to ringelmann (dataset). doi: 10.5281/zenodo.14831.

## Appendix A. Descriptive statistics of data (N=24572)

**Table:**

Variables (Mean, Median)	Standardized	Non-standardized
Individual productivity		
Mean (SD)	0.00 (1.00)	12700 (63300)
Median [Min, Max]	-0.195 [-0.201, 15.9]	384 [0, 1020000]
Individual-level clustering (Weighted)		
Mean (SD)	0.00 (1.00)	0.854 (0.242)
Median [Min, Max]	0.603 [-3.53, 0.603]	1.00 [0, 1.00]
Individual-level degree (Weighted)		
Mean (SD)	0.00 (1.00)	11200 (198000)
Median [Min, Max]	-0.0559 [-0.0564, 93.1]	93.0 [1.00, 18400000]
Individual-level constraint (Weighted)		
Mean (SD)	0.00 (1.00)	0.422 (0.252)
Median [Min, Max]	-0.269 [-1.50, 4.35]	0.354 [0.0440, 1.52]
Individual-level betweenness centrality (Weighted)		
Mean (SD)	0.00 (1.00)	809 (8680)
Median [Min, Max]	-0.0826 [-0.0932, 111]	91.9 [0, 966000]
Individual-level closeness centrality (Weighted)		
Mean (SD)	0.00 (1.00)	0.00262 (0.0386)
Median [Min, Max]	-0.0526 [-0.0674, 25.8]	0.000590 [0.0000157, 1.00]
Individual-level eigenvector centrality (Weighted)		
Mean (SD)	0.00 (1.00)	0.0166 (0.0832)
Median [Min, Max]	-0.193 [-0.200, 11.8]	0.000532 [0, 1.00]
Network-level clustering (Weighted)		
Mean (SD)	0.00 (1.00)	0.876 (0.0295)
Median [Min, Max]	-0.201 [-6.14, 3.37]	0.870 [0.695, 0.975]
Network-level transitivity (Weighted)		
Mean (SD)	0.00 (1.00)	0.366 (0.159)
Median [Min, Max]	-0.0637 [-1.39, 3.70]	0.356 [0.144, 0.955]
Network-level betweenness centralisation (Weighted)		
Mean (SD)	0.00 (1.00)	0.194 (0.130)
Median [Min, Max]	0.136 [0.0257, 0.821]	0.136 [0.0257, 0.821]
Network-level closeness centralisation (Weighted)		
Mean (SD)	0.00 (1.00)	0.859 (0.132)
Median [Min, Max]	0.144 [-4.17, 2.12]	0.878 [0.307, 1.14]
Network-level eigenvector centralisation (Weighted)		
Mean (SD)	0.00 (1.00)	0.790 (0.123)
Median [Min, Max]	0.125 [-4.99, 1.26]	0.805 [0.176, 0.944]
Network-level degree centralisation (Weighted)		
Mean (SD)	0.00 (1.00)	0.786 (0.119)
Median [Min, Max]	0.414 [-5.22, 1.30]	0.835 [0.167, 0.940]

## Appendix B: multilevel regression models equations (Individual productivity models)

Intercept-only model with only individual-level effects.

1 $$Y_{{ij}} = {\text{ }}\beta _{0} {\text{ }} + {\text{ }}\varepsilon _{{ij}}$$

Intercept-only model with mixed-effects: individual- and project-level as random effects.

2 $$Y_{{ij}} = {\text{ }}\beta _{{0{\text{ }}}} + {\text{ }}\gamma _{j} + {\text{ }}\varepsilon _{{ij}}$$

Base model 1 with all individual-level variables.

3 $$\begin{aligned} Y_{{ij}} & = {\text{ }}\beta _{0} {\text{ }} + {\text{ }}\beta _{1} Cluster_{{ij}} + {\text{ }}\beta _{2} Degree_{{ij}} \\ & + {\text{ }}\beta _{3} Constra\text{int} _{{ij}} + {\text{ }}\beta _{4} Betweenness\_Centrality_{{ij}} \\ & + {\text{ }}\beta _{5} Closeness\_Centrality_{{ij}} \\ & + {\text{ }}\beta _{6} Eigenvector\_Centrality_{{ij}} + {\text{ }}\gamma _{j} + {\text{ }}\varepsilon _{{ij}} \\ \end{aligned}$$

Base model 2 with individual- and network-level variables.

4 $$\begin{aligned} Y_{{ij}} & = {\text{ }}\beta _{0} {\text{ }} + {\text{ }}\beta _{1} Cluster_{{ij}} + {\text{ }}\beta _{2} Degree_{{ij}} \\ & + {\text{ }}\beta _{3} Constra\text{int} _{{ij}} + {\text{ }}\beta _{4} Betweenness\_Centrality_{{ij}} \\ & + {\text{ }}\beta _{5} Closeness\_Centrality_{{ij}} + {\text{ }}\beta _{6} Eigenvector\_Centrality_{{ij}} ~ \\ & + {\text{ }}\beta _{8} \Pr oject\_Clustering_{j} + {\text{ }}\beta _{9} \Pr oject\_Transitivity_{j} \\ & + {\text{ }}\beta _{{10}} \Pr oject\_Betweenness\_Centralisation_{j} \\ & + {\text{ }}\beta _{{11}} \Pr oject\_Closeness\_Centralisation_{j} \\ & + {\text{ }}\beta _{{12}} \Pr oject\_Degree\_Centralisation_{j} + {\text{ }}\gamma _{j} + {\text{ }}\varepsilon _{{ij}} \\ \end{aligned}$$

Base model 3 with individual- and network-level variables.

5 $$\begin{aligned} Y_{{ij}} & = {\text{ }}\beta _{0} {\text{ }} + {\text{ }}\beta _{1} Cluster_{{ij}} + {\text{ }}\beta _{2} Degree_{{ij}} \\ & + {\text{ }}\beta _{3} Constra\text{int} _{{ij}} + {\text{ }}\beta _{4} Betweenness\_Centrality_{{ij}} \\ & + {\text{ }}\beta _{5} Closeness\_Centrality_{{ij}} + {\text{ }}\beta _{6} Eigenvector\_Centrality_{{ij}} ~ \\ & + {\text{ }}\beta _{8} \Pr oject\_Clustering_{j} + {\text{ }}\beta _{{10}} \Pr oject\_Betweenness\_Centralisation_{j} \\ & + {\text{ }}\beta _{{11}} \Pr oject\_Closeness\_Centralisation_{j} \\ & + {\text{ }}\beta _{{12}} \Pr oject\_Degree\_Centralisation_{j} \\ & + {\text{ }}\beta _{{12}} \Pr oject\_Eigenvector\_Centralisation_{j} + {\text{ }}\gamma _{j} + {\text{ }}\varepsilon _{{ij}} \\ \end{aligned}$$

Where:

- Y_ij_: Individual productivity for individual i in project j.
- β₀: Overall intercept, representing the average individual productivity across all projects.
- γ_j_: Random intercept for project j, capturing the deviation of project ’’s average individual productivity from the overall intercept. This accounts for potential differences in productivity levels between projects.
- ε_ij_: Individual-level error term, capturing the random variation in individual productivity not explained by the model.
- β₁- β_12_: Coefficient for SNP predictors.

Quadratic model with individual variables.

6 $$\begin{aligned} Yij & = \beta _{0} + \sum\nolimits_{{k = 1}}^{6} {\beta _{k} } X_{{k,ij}} + \beta _{k} 'X_{{k,ij}} ^{2} \\ & + \sum\nolimits_{{m = 7}}^{{11}} {\beta _{m} } Z_{{m,j}} + \gamma _{j} + \varepsilon _{{ij}} \\ \end{aligned}$$

Where:

- X_k, ij_​ = Individual SNPs.
- Z_m, j_= Project SNPs.
- β_k_′ = the coefficients for the quadratic terms of individual SNPs.
- γ_j_: Random intercept for project j, capturing the deviation of project ’’s average individual productivity from the overall intercept. This accounts for potential differences in productivity levels between projects.
- ε_ij_: Individual-level error term, capturing the random variation in individual productivity not explained by the model.

Interaction models with individual-network SNP interaction variables.

7 $$\begin{aligned} Y_{{ij}} & = {\text{ }}\beta _{0} {\text{ }} + {\text{ }}\beta _{1} Cluster_{{ij}} + {\text{ }}\beta _{2} Degree_{{ij}} \\ & + {\text{ }}\beta _{3} Constra\text{int} _{{ij}} + {\text{ }}\beta _{4} Betweenness\_Centrality_{{ij}} \\ & + {\text{ }}\beta _{5} Closeness\_Centrality_{{ij}} + {\text{ }}\beta _{6} Eigenvector\_Centrality_{{ij}} ~ \\ & + {\text{ }}\beta _{8} \Pr oject\_Clustering_{j} + {\text{ }}\beta _{{10}} \Pr oject\_Betweenness\_Centralisation_{j} \\ & + {\text{ }}\beta _{{11}} \Pr oject\_Closeness\_Centralisation_{j} \\ & + {\text{ }}\beta _{{12}} \Pr oject\_Degree\_Centralisation_{j} \\ & + {\text{ }}\beta _{{12}} \Pr oject\_Eigenvector\_Centralisation_{j} \\ & + {\text{ }}\beta _{k} X_{{kij}} Z_{{kj}} {\text{ }} + {\text{ }}\gamma _{j} + {\text{ }}\varepsilon _{{ij}} \\ \end{aligned}$$

Where:

- Y_ij_: Individual productivity for individual i in project j.
- β₀: Overall intercept, representing the average individual productivity across all projects.
- γ_j_: Random intercept for project j, capturing the deviation of project ’’s average individual productivity from the overall intercept. This accounts for potential differences in productivity levels between projects.
- ε_ij_: Individual-level error term, capturing the random variation in individual productivity not explained by the model.
- β₁- β_12_: Coefficient for SNP predictors.
- X_kij_ : Individual-level predictors (e.g., Degree_ij_, Betweenness_Centrality_ij_).
- Z_kj_ : Network-level predictors (e.g., Project_Clustering_j_, Project_Degree_Centralisation_j_).
- k : the index of the pairs of variables considered for interaction.
- X_kij_Z_k_ : the interaction pairs does not include the pairs where the variables are from the same category, e.g. Individual cluster * network clustering, individual betweenness centrality * network betweenness Centralisation.

## Appendix C1. Results of individual SNP quadratic models

**Table:**

	Individual SNP quadratic models									
	Model 1		Model 2		Model 3		Model 4	Model 5		Model 6
(Intercept)	0.15		0.07		0.08		0.08	0.07		0.15
	(0.08)		(0.08)		(0.08)		(0.08)	(0.08)		(0.08)
Individual clustering	-0.39***		-0.27***		-0.28***		-0.24***	-0.27***		-0.22***
	(0.01)		(0.01)		(0.01)		(0.01)	(0.01)		(0.01)
Individual degree	0.01		0.06***		0.01		0.01	0.01		0.01
	(0.01)		(0.01)		(0.01)		(0.01)	(0.01)		(0.01)
Individual constraint	-0.08***		-0.13***		-0.10***		-0.12***	-0.13***		-0.11***
	(0.01)		(0.01)		(0.01)		(0.01)	(0.01)		(0.01)
Individual betweenness centrality	0.04***		0.03***		0.03***		0.18***	0.03***		0.03***
	(0.01)		(0.01)		(0.01)		(0.01)	(0.01)		(0.01)
Individual closeness centrality	-0.01*		-0.02***		-0.02***		-0.02***	-0.04		-0.01*
	(0.01)		(0.01)		(0.01)		(0.01)	(0.03)		(0.01)
Individual eigenvector centrality	0.13***		0.12***		0.12***		0.13***	0.12***		0.54***
	(0.01)		(0.01)		(0.01)		(0.01)	(0.01)		(0.02)
Network clustering	0.01		0.01		0.01		0.00	0.01		0.00
	(0.04)		(0.04)		(0.04)		(0.04)	(0.04)		(0.04)
Network betweenness centralisation	-0.34		-0.20		-0.03		-0.25	-0.21		-0.41
	(0.31)		(0.31)		(0.32)		(0.31)	(0.31)		(0.31)
Network closeness centralisation	0.01		0.01		0.01		0.01	0.01		-0.01
	(0.05)		(0.05)		(0.05)		(0.05)	(0.05)		(0.05)
Network degree centralisation	0.10*		0.12**		0.12**		0.11*	0.12**		0.14**
	(0.04)		(0.04)		(0.05)		(0.04)	(0.04)		(0.04)
Network eigenvector centralisation	-0.08		-0.11		-0.12*		-0.11	-0.11		-0.08
	(0.06)		(0.06)		(0.06)		(0.06)	(0.06)		(0.06)
(Individual clustering)^2^	-0.07***									
	(0.01)									
(Individual degree) ^2^			-0.00***							
			(0.00)							
(Individual constraint) ^2^					-0.04***					
					(0.01)					
(Individual betweenness centrality) ^2^							-0.00***			
							(0.00)			
(Individual coseness centrality) ^2^								0.00		
								(0.00)		
(Individual eigenvector centrality) ^2^										-0.04***
										(0.00)
AIC	64630.32		64759.34		64729.41		64574.02	64774.18		64200.25
BIC	64751.96		64880.99		64851.05		64695.66	64895.82		64321.89
Log Likelihood	-32300.16		-32364.67		-32349.71		-32272.01	-32372.09		-32085.13
Num. obs.	24,572		24,572		24,572		24,572	24,572		24,572
Num. groups: project_name	58		58		58		58	58		58
Var: project_name (Intercept)	0.09		0.09		0.09		0.09	0.09		0.09
Var: Residual	0.80		0.81		0.81		0.80	0.81		0.79
Marginal R^2^ / Conditional R^2^	0.13/0.22		0.13/0.22		0.13/0.22		0.14/0.22	0.13/0.22		0.15/0.24

****p* < 0.001; ***p* < 0.01; **p* < 0.05.

## Appendix C2. Results of network SNP quadratic models

**Table:**

	Network SNP quadratic models					
	Model 7	Model 8	Model 9	Model 10	Model 11	Model 12
Intercept	0.06	0.02	0.07	0.07	0.07	0.11
	(0.08)	(0.14)	(0.08)	(0.08)	(0.08)	(0.08)
Individual clustering	-0.27^***^	-0.27^***^	-0.27^***^	-0.27^***^	-0.27^***^	-0.27^***^
	(0.01)	(0.01)	(0.01)	(0.01)	(0.01)	(0.01)
Individual degree	0.01	0.01	0.01	0.01	0.01	0.01
	(0.01)	(0.01)	(0.01)	(0.01)	(0.01)	(0.01)
Individual constraint	-0.13^***^	-0.13^***^	-0.13^***^	-0.13^***^	-0.13^***^	-0.13^***^
	(0.01)	(0.01)	(0.01)	(0.01)	(0.01)	(0.01)
Individual betweenness centrality	0.03^***^	0.03^***^	0.03^***^	0.03^***^	0.03^***^	0.03^***^
	(0.01)	(0.01)	(0.01)	(0.01)	(0.01)	(0.01)
Individual closeness centrality	-0.02^***^	-0.02^***^	-0.02^***^	-0.02^***^	-0.02^***^	-0.02^***^
	(0.01)	(0.01)	(0.01)	(0.01)	(0.01)	(0.01)
Individual eigenvector centrality	0.12^***^	0.12^***^	0.12^***^	0.12^***^	0.12^***^	0.12^***^
	(0.01)	(0.01)	(0.01)	(0.01)	(0.01)	(0.01)
Network clustering	-0.01	0.01	0.01	0.01	0.04	0.07
	(0.04)	(0.04)	(0.04)	(0.04)	(0.04)	(0.05)
Network betweenness centralisation	-0.10	0.26	-0.21	-0.12	-0.14	-0.20
	(0.32)	(0.95)	(0.32)	(0.31)	(0.31)	(0.31)
Network closeness centralisation	-0.00	0.01	0.01	0.00	-0.00	-0.04
	(0.05)	(0.05)	(0.05)	(0.05)	(0.05)	(0.04)
Network degree centralisation	0.11^*^	0.12^**^	0.12	0.07	0.09	0.07
	(0.04)	(0.04)	(0.06)	(0.05)	(0.05)	(0.05)
Network eigenvector centralisation	-0.12^*^	-0.12^*^	-0.11	-0.13^*^	-0.17^*^	
	(0.06)	(0.06)	(0.06)	(0.06)	(0.07)	
(Network clustering) ^2^	-0.01					
	(0.01)					
(Network betweenness centralisation) ^2^		-0.67				
		(1.27)				
(Network closeness centralisation) ^2^			-0.00			
			(0.03)			
(Network degree centralisation) ^2^				-0.03		
				(0.02)		
(Network eigenvector centralisation) ^2^					-0.03	
					(0.02)	
Network transitivity						0.16^*^
						(0.07)
(Network transitivity) ^2^						-0.06
						(0.04)
AIC	64768.11	64760.13	64767.99	64766.74	64766.52	64765.81
BIC	64889.75	64881.77	64889.64	64888.38	64888.16	64887.45
Log Likelihood	-32369.05	-32365.07	-32369.00	-32368.37	-32368.26	-32367.91
Num. obs.	24,572	24,572	24,572	24,572	24,572	24,572
Num. groups: project_name	58	58	58	58	58	58
Var: project_name (Intercept)	0.09	0.09	0.09	0.09	0.09	0.09
Var: Residual	0.81	0.81	0.81	0.81	0.81	0.81
Marginal R^2^ / Conditional R^2^	0.13/0.22	0.13/0.22	0.13/0.22	0.13/0.22	0.13/0.22	0.13/0.22

^***^*p* < 0.001; ^**^*p* < 0.01; ^*^*p* < 0.05

.

## Appendix D1. Interaction effects between individual and network predictors (Model 1–8)

**Table:**

	Model 1	Model 2	Model 3	Model 4	Model 5	Model 6	Model 7	Model 8
Intercept	0.01	0.07	0.08	0.10	0.11	0.09	0.07	0.07
	(0.08)	(0.08)	(0.08)	(0.08)	(0.08)	(0.08)	(0.08)	(0.08)
Individual clustering	-0.40^***^	-0.28^***^	-0.27^***^	-0.29^***^	-0.28^***^	-0.27^***^	-0.27^***^	-0.27^***^
	(0.01)	(0.01)	(0.01)	(0.01)	(0.01)	(0.01)	(0.01)	(0.01)
Individual degree	0.01	0.01	0.01	0.01	0.01	0.27^***^	0.02	-0.00
	(0.01)	(0.01)	(0.01)	(0.01)	(0.01)	(0.03)	(0.01)	(0.01)
Individual constraint	-0.12^***^	-0.14^***^	-0.13^***^	-0.12^***^	-0.12^***^	-0.13^***^	-0.13^***^	-0.13^***^
	(0.01)	(0.01)	(0.01)	(0.01)	(0.01)	(0.01)	(0.01)	(0.01)
Individual betweenness centrality	0.04^***^	0.03^***^	0.03^***^	0.06^***^	0.05^***^	0.03^***^	0.03^***^	0.03^***^
	(0.01)	(0.01)	(0.01)	(0.01)	(0.01)	(0.01)	(0.01)	(0.01)
Individual closeness centrality	-0.02^**^	-0.01	-0.02^**^	-0.02^***^	-0.03^***^	-0.02^***^	-0.02^***^	-0.02^***^
	(0.01)	(0.01)	(0.01)	(0.01)	(0.01)	(0.01)	(0.01)	(0.01)
Individual egenvector centrality	0.11^***^	0.12^***^	0.13^***^	0.10^***^	0.11^***^	0.12^***^	0.12^***^	0.12^***^
	(0.01)	(0.01)	(0.01)	(0.01)	(0.01)	(0.01)	(0.01)	(0.01)
Network clustering	-0.00	0.03	-0.00	0.04	0.02	0.01	0.01	0.00
	(0.04)	(0.04)	(0.04)	(0.04)	(0.04)	(0.04)	(0.04)	(0.04)
Network betweenness centralisation	0.10	-0.16	-0.22	-0.33	-0.34	-0.33	-0.21	-0.21
	(0.30)	(0.31)	(0.31)	(0.31)	(0.31)	(0.31)	(0.31)	(0.31)
Network bloseness bentralisation	0.01	0.03	0.01	0.02	-0.02	0.02	0.01	0.01
	(0.05)	(0.05)	(0.05)	(0.05)	(0.04)	(0.05)	(0.05)	(0.05)
Network degree centralisation	0.12^**^	0.11^*^	0.11^**^	0.10^*^	0.11^*^	0.12^**^	0.12^**^	0.12^**^
	(0.04)	(0.04)	(0.04)	(0.04)	(0.05)	(0.04)	(0.04)	(0.04)
Network eigenvector centralisation	-0.13^*^	-0.13^*^	-0.10	-0.12^*^		-0.11	-0.11	-0.11
	(0.06)	(0.06)	(0.06)	(0.06)		(0.06)	(0.06)	(0.06)
Individual clustering * Network betweenness centralisation	0.56^***^							
	(0.04)							
Individual clustering * Network closeness centralisation		0.08^***^						
		(0.01)						
Individual clustering * Network degree centralisation			-0.03^***^					
			(0.01)					
Individual clustering * Network eigenvector centralisation				0.15^***^				
				(0.01)				
Network transitivity					0.09			
					(0.06)			
Individual clustering * Network transitivity					-0.08^***^			
					(0.01)			
Individual Degree * Network Betweenness Centralisation						-2.41^***^		
						(0.31)		
Individual degree * Network closeness centralisation							0.01	
							(0.01)	
Individual degree * Network eigenvector centralisation								-0.03^***^
								(0.01)
AIC	64560.25	64550.25	64751.78	64322.97	64624.53	64704.72	64769.37	64756.58
BIC	64681.89	64671.89	64873.42	64444.61	64746.17	64826.36	64891.01	64878.22
Log Likelihood	-32265.12	-32260.12	-32360.89	-32146.49	-32297.26	-32337.36	-32369.69	-32363.29
Num. obs.	24,572	24,572	24,572	24,572	24,572	24,572	24,572	24,572
Num. groups: project_name	58	58	58	58	58	58	58	58
Var: project_name (Intercept)	0.08	0.09	0.09	0.09	0.09	0.09	0.09	0.09
Var: Residual	0.80	0.80	0.81	0.79	0.80	0.81	0.81	0.81
Marginal R^2^ / Conditional R^2^	0.14/0.22	0.14/0.23	0.13/0.22	0.14/0.23	0.14/0.22	0.13/0.22	0.13/0.22	0.13/0.22

^***^*p* < 0.001; ^**^*p* < 0.01; ^*^*p* < 0.05

.

## Appendix D2. Interaction effects between individual and network predictors (Model 9–16)

**Table:**

	Model 9	Model 10	Model 11	Model 12	Model 13	Model 14	Model 15	Model 16
Intercept	0.07	0.09	0.03	0.07	0.07	0.08	0.10	0.10
	(0.08)	(0.08)	(0.08)	(0.08)	(0.08)	(0.08)	(0.09)	(0.08)
Individual clustering	-0.27^***^	-0.27^***^	-0.27^***^	-0.27^***^	-0.27^***^	-0.27^***^	-0.27^***^	-0.27^***^
	(0.01)	(0.01)	(0.01)	(0.01)	(0.01)	(0.01)	(0.01)	(0.01)
Individual degree	0.02	-0.02	0.01	0.01	0.01	0.01	0.01	0.01
	(0.02)	(0.01)	(0.01)	(0.01)	(0.01)	(0.01)	(0.01)	(0.01)
Individual constraint	-0.13^***^	-0.13^***^	-0.07^***^	-0.13^***^	-0.13^***^	-0.13^***^	-0.13^***^	-0.13^***^
	(0.01)	(0.01)	(0.02)	(0.01)	(0.01)	(0.01)	(0.01)	(0.01)
Individual betweenness centrality	0.03^***^	0.03^***^	0.03^***^	0.03^***^	0.03^***^	0.03^***^	0.03^***^	0.03^***^
	(0.01)	(0.01)	(0.01)	(0.01)	(0.01)	(0.01)	(0.01)	(0.01)
Individual closeness centrality	-0.02^***^	-0.02^***^	-0.02^***^	-0.03^***^	-0.02^***^	-0.02^***^	-0.02^***^	-0.02^***^
	(0.01)	(0.01)	(0.01)	(0.01)	(0.01)	(0.01)	(0.01)	(0.01)
Individual eigenvector centrality	0.12^***^	0.12^***^	0.12^***^	0.12^***^	0.12^***^	0.12^***^	0.12^***^	0.13^***^
	(0.01)	(0.01)	(0.01)	(0.01)	(0.01)	(0.01)	(0.01)	(0.01)
Network clustering	0.01	0.00	0.00	-0.01	0.00	0.01	0.05	0.01
	(0.04)	(0.04)	(0.04)	(0.03)	(0.04)	(0.04)	(0.04)	(0.04)
Network betweenness centralisation	-0.21	-0.27	0.09	-0.28	-0.21	-0.24	-0.33	-0.31
	(0.31)	(0.31)	(0.32)	(0.30)	(0.31)	(0.31)	(0.32)	(0.31)
Network closeness centralisation	0.01	-0.01	0.01	0.00	0.01	0.01	0.02	-0.02
	(0.05)	(0.04)	(0.05)	(0.05)	(0.05)	(0.05)	(0.05)	(0.04)
Network degree centralisation	0.12^**^	0.12^**^	0.13^**^	0.10^*^	0.11^*^	0.12^**^	0.13^**^	0.12^*^
	(0.04)	(0.05)	(0.04)	(0.04)	(0.04)	(0.04)	(0.05)	(0.05)
Network eigenvector centralisation	-0.11		-0.13^*^	-0.11^*^	-0.11	-0.11	-0.11	
	(0.06)		(0.06)	(0.06)	(0.06)	(0.06)	(0.06)	
Individual degree * Network clustering	0.01							
	(0.02)							
Network transitivity		0.10						0.09
		(0.06)						(0.06)
Individual degree * Network transitivity		0.05^**^						
		(0.01)						
Individual constraint * Network betweenness centralisation			-0.26^***^					
			(0.06)					
Individual constraint * Network closeness centralisation				0.06^***^				
				(0.01)				
Individual constraint * Network degree centralisation					0.01			
					(0.01)			
Individual constraint * Network eigenvector centralisation						-0.01		
						(0.01)		
Individual constraint * Network clustering							-0.05^***^	
							(0.01)	
Individual constraint * Network transitivity								0.02
								(0.01)
AIC	64768.55	64760.49	64748.49	64716.12	64768.41	64769.42	64738.97	64768.20
BIC	64890.19	64882.13	64870.14	64837.76	64890.05	64891.06	64860.61	64889.84
Log Likelihood	-32369.28	-32365.24	-32359.25	-32343.06	-32369.21	-32369.71	-32354.49	-32369.10
Num. obs.	24,572	24,572	24,572	24,572	24,572	24,572	24,572	24,572
Num. groups: project_name	58	58	58	58	58	58	58	58
Var: project_name (Intercept)	0.09	0.09	0.09	0.08	0.09	0.09	0.10	0.09
Var: Residual	0.81	0.81	0.81	0.81	0.81	0.81	0.81	0.81
Marginal R^2^ / Conditional R^2^	0.13/0.22	0.13/0.22	0.13/0.22	0.14/0.22	0.13/0.22	0.13/0.22	0.13/0.23	0.13/0.22

^***^*p* < 0.001; ^**^*p* < 0.01; ^*^*p* < 0.05

.

## Appendix D3. Interaction effects between individual and network predictors (Model 17–24)

**Table:**

	Model 17	Model 18	Model 19	Model 20	Model 21		Model 22	Model 23	Model 24
Intercept	0.07	0.07	0.10	0.08	0.11		0.07	0.07	0.07
	(0.08)	(0.08)	(0.08)	(0.08)	(0.08)		(0.08)	(0.08)	(0.08)
Individual clustering	-0.27^***^	-0.27^***^	-0.23^***^	-0.26^***^	-0.24^***^		-0.27^***^	-0.27^***^	-0.27^***^
	(0.01)	(0.01)	(0.01)	(0.01)	(0.01)		(0.01)	(0.01)	(0.01)
Individual degree	0.01	0.01	0.00	0.01	0.01		0.01	0.01	0.01
	(0.01)	(0.01)	(0.01)	(0.01)	(0.01)		(0.01)	(0.01)	(0.01)
Individual constraint	-0.13^***^	-0.13^***^	-0.11^***^	-0.13^***^	-0.12^***^		-0.13^***^	-0.13^***^	-0.13^***^
	(0.01)	(0.01)	(0.01)	(0.01)	(0.01)		(0.01)	(0.01)	(0.01)
Individual betweenness centrality	0.03^***^	0.02	0.69^***^	0.09^***^	0.44^***^		0.03^***^	0.03^***^	0.03^***^
	(0.01)	(0.01)	(0.04)	(0.01)	(0.03)		(0.01)	(0.01)	(0.01)
Individual closeness centrality	-0.02^***^	-0.02^***^	-0.02^**^	-0.02^***^	-0.02^***^		-0.02	-0.03^*^	-0.02^***^
	(0.01)	(0.01)	(0.01)	(0.01)	(0.01)		(0.01)	(0.01)	(0.01)
Individual eigenvector centrality	0.12^***^	0.12^***^	0.13^***^	0.13^***^	0.13^***^		0.12^***^	0.12^***^	0.12^***^
	(0.01)	(0.01)	(0.01)	(0.01)	(0.01)		(0.01)	(0.01)	(0.01)
Network clustering	0.01	0.01	0.00	0.00	0.01		0.01	0.01	0.01
	(0.04)	(0.04)	(0.04)	(0.04)	(0.04)		(0.04)	(0.04)	(0.04)
Network betweenness centralisation	-0.21	-0.21	-0.20	-0.21	-0.27		-0.21	-0.21	-0.21
	(0.31)	(0.31)	(0.31)	(0.31)	(0.31)		(0.31)	(0.31)	(0.31)
Network closeness centralisation	0.01	0.01	0.01	0.01	-0.02		0.01	0.01	0.01
	(0.05)	(0.05)	(0.05)	(0.05)	(0.04)		(0.05)	(0.05)	(0.05)
Network degree centralisation	0.12^**^	0.12^**^	0.10^*^	0.12^**^	0.11^*^		0.12^**^	0.12^**^	0.12^**^
	(0.04)	(0.04)	(0.04)	(0.04)	(0.05)		(0.04)	(0.04)	(0.04)
Network eigenvector centralisation	-0.11	-0.11	-0.15^**^	-0.11			-0.11	-0.11	-0.11
	(0.06)	(0.06)	(0.06)	(0.06)			(0.06)	(0.06)	(0.06)
Individual betweenness centrality * Network closeness centralisation	-0.00								
	(0.02)								
Individual betweenness centrality * Network degree centralisation		0.04							
		(0.03)							
Individual betweenness centrality * Network eigenvector centralisation			-0.54^***^						
			(0.03)						
Individual betweenness centrality * Network clustering				-0.05^***^					
				(0.01)					
Network transitivity					0.11^*^				
					(0.06)				
Individual betweenness centrality * Network transitivity					0.30^***^				
					(0.03)				
Individual closeness centrality * Network betweenness centralisation							-0.02		
							(0.04)		
Individual closeness centrality * Network degree centralisation								-0.00	
								(0.01)	
Individual closeness centrality * Network eigenvector centralisation									0.00
									(0.01)
AIC	64769.06	64766.04	64506.60	64750.82	64628.27		64767.33	64770.53	64770.01
BIC	64890.70	64887.68	64628.24	64872.46	64749.91		64888.97	64892.17	64891.65
Log Likelihood	-32369.53	-32368.02	-32238.30	-32360.41	-32299.14		-32368.67	-32370.27	-32370.00
Num. obs.	24,572	24,572	24,572	24,572	24,572		24,572	24,572	24,572
Num. groups: project_name	58	58	58	58	58		58	58	58
Var: project_name (Intercept)	0.09	0.09	0.09	0.09	0.09		0.09	0.09	0.09
Var: Residual	0.81	0.81	0.80	0.81	0.80		0.81	0.81	0.81
Marginal R^2^ / Conditional R^2^	0.13/0.22	0.13/0.22	0.14/0.22	0.13/0.22	0.14/0.22		0.13/0.22	0.13/0.22	0.13/0.22

^***^*p* < 0.001; ^**^*p* < 0.01; ^*^*p* < 0.05

.

## Appendix D4. Interaction effects between individual and network predictors (Model 25–31)

**Table:**

	Model 25	Model 26	Model 27	Model 28	Model 29	Model 30	Model 31
Intercept	0.07	0.09	0.06	0.08	0.07	0.08	0.09
	(0.08)	(0.08)	(0.08)	(0.08)	(0.08)	(0.08)	(0.08)
Individual clustering	-0.27^***^	-0.27^***^	-0.27^***^	-0.27^***^	-0.27^***^	-0.27^***^	-0.27^***^
	(0.01)	(0.01)	(0.01)	(0.01)	(0.01)	(0.01)	(0.01)
Individual degree	0.01	0.01	0.01	0.01	0.01	0.01	0.01
	(0.01)	(0.01)	(0.01)	(0.01)	(0.01)	(0.01)	(0.01)
Individual constraint	-0.13^***^	-0.13^***^	-0.13^***^	-0.13^***^	-0.13^***^	-0.13^***^	-0.13^***^
	(0.01)	(0.01)	(0.01)	(0.01)	(0.01)	(0.01)	(0.01)
Individual betweenness centrality	0.03^***^	0.03^***^	0.04^***^	0.03^***^	0.03^***^	0.03^***^	0.03^***^
	(0.01)	(0.01)	(0.01)	(0.01)	(0.01)	(0.01)	(0.01)
Individual closeness centrality	-0.02^***^	-0.02^***^	-0.02^***^	-0.02^***^	-0.02^***^	-0.02^***^	-0.02^***^
	(0.01)	(0.01)	(0.01)	(0.01)	(0.01)	(0.01)	(0.01)
Individual eigenvector centrality	0.12^***^	0.12^***^	0.15^***^	0.12^***^	0.13^***^	0.12^***^	0.12^***^
	(0.01)	(0.01)	(0.01)	(0.01)	(0.01)	(0.01)	(0.01)
Network clustering	0.01	0.01	0.00	0.01	0.00	0.01	0.01
	(0.04)	(0.04)	(0.04)	(0.04)	(0.04)	(0.04)	(0.04)
Network betweenness centralisation	-0.21	-0.27	-0.13	-0.22	-0.19	-0.23	-0.27
	(0.31)	(0.31)	(0.31)	(0.31)	(0.31)	(0.31)	(0.31)
Network closeness centralisation	0.01	-0.02	0.01	0.02	0.01	0.01	-0.02
	(0.05)	(0.04)	(0.05)	(0.05)	(0.05)	(0.05)	(0.04)
Network degree centralisation	0.12^**^	0.12^*^	0.12^**^	0.12^**^	0.11^*^	0.12^**^	0.12^*^
	(0.04)	(0.05)	(0.04)	(0.04)	(0.04)	(0.04)	(0.05)
Network eigenvector centralisation	-0.11		-0.11^*^	-0.10	-0.12^*^	-0.11	
	(0.06)		(0.06)	(0.06)	(0.06)	(0.06)	
Individual closeness centrality * Network clustering	-0.00						
	(0.00)						
Network transitivity		0.09					0.09
		(0.06)					(0.06)
Individual closeness centrality * Network transitivity		-0.00					
		(0.01)					
Individual eigenvector centrality * Network betweenness centralisation			-0.16^***^				
			(0.04)				
Individual eigenvector centrality * Network closeness centralisation				-0.02^***^			
				(0.00)			
Individual eigenvector centrality * Network degree centralisation					0.01^**^		
					(0.00)		
Individual eigenvector centrality * Network clustering						-0.01	
						(0.01)	
Individual eigenvector centrality * Network transitivity							0.01
							(0.01)
AIC	64771.62	64771.77	64754.46	64759.29	64763.37	64769.16	64771.23
BIC	64893.26	64893.41	64876.10	64880.93	64885.01	64890.80	64892.87
Log Likelihood	-32370.81	-32370.88	-32362.23	-32364.64	-32366.69	-32369.58	-32370.61
Num. obs.	24,572	24,572	24,572	24,572	24,572	24,572	24,572
Num. groups: project_name	58	58	58	58	58	58	58
Var: project_name (Intercept)	0.09	0.09	0.09	0.09	0.09	0.09	0.09
Var: Residual	0.81	0.81	0.81	0.81	0.81	0.81	0.81
Marginal R^2^ / Conditional R^2^	0.13/0.22	0.13/0.22	0.13/0.22	0.13/0.22	0.13/0.22	0.13/0.22	0.13/0.22

^***^*p* < 0.001; ^**^*p* < 0.01; ^*^*p* < 0.05.

## Abbreviations

OSSD: Open source software development
SNP: Social network property
VIF: Variance inflation factor
